# Survival and relapse patterns in patients of cranial vs extra-cranial oligometastases treated with stereotactic radiosurgery/stereotactic body radiation therapy and systemic therapy

**DOI:** 10.1093/bjro/tzae042

**Published:** 2024-11-27

**Authors:** Anil Kumar Anand, Neha Kakkar, Vivek Immanuel, Jyoti Pannu, Amal Roy Chaudhoory, Heigrujam Malhotra, Tarun Kumar

**Affiliations:** Department of Radiation Oncology, Fortis Memorial Research Institute, Gurugram, Haryana 122002, India; Department of Radiation Oncology, Fortis Memorial Research Institute, Gurugram, Haryana 122002, India; Department of Radiation Oncology, Fortis Memorial Research Institute, Gurugram, Haryana 122002, India; Department of Radiation Oncology, Fortis Memorial Research Institute, Gurugram, Haryana 122002, India; Department of Radiation Oncology, Fortis Memorial Research Institute, Gurugram, Haryana 122002, India; Division of Medical Physics, Fortis Memorial Research Institute, Gurugram, Haryana 122002, India; Division of Medical Physics, Fortis Memorial Research Institute, Gurugram, Haryana 122002, India

**Keywords:** SBRT/SRS, cranial/extra cranial, oligometastases

## Abstract

**Objectives:**

To evaluate the outcome of patients with cranial (C) and extra-cranial (EC) oligometastases treated with stereotactic radiosurgery (SRS)/stereotactic body radiotherapy (SBRT) and standard of care systemic therapy.

**Methods:**

During the period 2018-2022, patients who received SBRT or SRS for oligometastases (≤5 lesions) in addition to systemic therapy were evaluated. PET-CT was done to categorize them as C or EC oligometastases. Local control, distant progression, progression-free survival (PFS), overall survival (OS), and toxicity of the treatment were recorded.

**Results:**

43 patients received SBRT/SRS to 88 oligometastatic lesions. Eighteen patients had C metastases, 23 had EC metastases and 2 patients had both. 40/43 patients had received systemic therapy. At a median follow-up of 13 months, median PFS was 14 months and 1 and 2 years OS was 83.2% and 67.4%. Local control with SRS was 92.8% and with SBRT was 86.3%. Distant failure in C vs EC oligometastases was seen in 12/14 vs 7/20 patients (*P* = 0.03). Median PFS was 30 months for EC and 6 months for C oligometastases (*P* = 0.003). 1 and 2 years OS was 89.6% and 82.7% for EC and 77.6% and 48.5% for C oligometastases (*P* = 0.21). One patient had grade 3 and 3 patients had grade 1 toxicity.

**Conclusions:**

SRS and SBRT yielded high rates of local control with low toxicity. Compared to EC, patients with C oligometastases had higher distant relapses, poorer PFS, and a trend towards worse survival. More studies with separate enrolment of patients with C and EC oligometastases are needed.

**Advances in knowledge:**

Outcome of patients with C oligometastases is poorer than EC metastases and hence the studies should be separately done in these 2 groups to assess the benefit of SRS/SBRT.

## Introduction 

It has long been postulated that there is an intermediate stage of cancer between a localized disease and diffusely metastatic disease called oligometastatic disease (OMD).^1^ Oligometastatic state can be defined as the presence of 1-5 metastatic lesions which can be safely treated with aggressive local therapy.^2^ Synchronous oligometastases is defined as oligometastases present at the time of initial diagnosis while oligo recurrence is defined as growth of metastatic sites while the primary tumour is controlled for at least 3 months.^2^^,^^3^

Treating oligometastases with local modalities like surgery or stereotactic body radiotherapy (SBRT) in addition to systemic therapy can improve the patients’ outcome.^4–6^ It is also reported that oligometastatic cancer has a unique molecular signature and has better outcome as compared to diffusely metastatic disease.^6^

Stereotactic radiosurgery (SRS) and/or SBRT are specialized forms of radiation techniques characterized by delivery of high dose of radiation in 1-8 fractions with an accurate tumour targeting system yielding local control rates of 70%-95%.^7–9^

Most of the studies on SRS for patients with brain metastases have not reported exact status of the presence or absence of extra-cranial (EC) metastases.^10^^,^^11^ Similarly studies on SBRT for patients with EC metastases have either excluded or included few patients with brain metastases.^4^^,^^9^

Patients with cranial (C) oligometastases are likely to have different outcomes since most of the chemotherapy drugs do not cross the blood-brain barrier (BBB) and would have no or minimal effect on C metastases.^12^ There may also be differences in sites of further progression and survival outcomes.

Therefore, it is pertinent to evaluate whether approach to patients with C and EC oligometastases should be the same or different in terms of aggressively treating the local site with ablative treatments like SRS, SBRT, or surgery in addition to systemic therapy.

In this study, we have evaluated outcome of patients who received SRS/SBRT for C and EC metastases in addition to standard-of-care systemic therapy.

## Methods

In the period between January 2018 and December 2022, patients who received SBRT or SRS for OMD were screened. The study was approved by the ethics committee of the hospital.

Inclusion criteria included patients 18 years or older with histologically confirmed primary malignancy with OMD, ECOG PS 0-1, and life expectancy >6 months. Patients with up to 5 oligo-metastasis or oligo recurrence were included in the study. All sites of metastatic disease were suitable to be treated with SRS/SBRT. There was a gap of 2 weeks before and after radiotherapy (RT) for chemotherapy and 1 week for immunotherapy (IT). Hormonal therapy continued during SBRT. Exclusion criteria included patients with (≥5) metastases, ascites, pleural effusion, leptomeningeal disease, and ECOG ≥2.

All patients had haematological investigations and contrast CT scan/MRI of the involved region. FDG PET-CT scan was done to confirm number and location of metastases. Brain and spine MRI was done for patients with brain and spine metastases.

### SRS/SBRT procedure

Patients were immobilized with a thermoplastic cast depending on the site to be treated. A 3 layered stereotactic mask was made for SRS cases. Planning CT scan with contrast was done on CT Simulator (Siemens healthcare GmBH). Gross Tumor Volume (GTV) was delineated with the help of fusion with MRI or PET-CT scan and a Planning Target Volume (PTV) margin of 2-5 mm was given for SBRT and 1-2 mm for SRS. Liver lesions were delineated with triple phase CT planning scan. Organ motion management for lung and liver lesions was done with active breath control or an Internal target volume (ITV) generated by 4DCT. For SRS and SBRT, target coverage included D95 of GTV of 95%-98% and PTV 80%-90% of the prescription dose. Hot spots within the GTV were acceptable up to 120%. Constraints for Organs at risk (OARs) were given as per TG101.^13^

Dose/fractionation of SRS and SBRT is depicted in Table 1. The patients were planned on Monaco 5.51 Treatment Planning System (Elekta AB, Stockholm, Sweden) using volumetric arc therapy and treated on “Elekta Axesse” linac with image guidance with cone beam CT and Exac-Trac 6D image guidance system.

**Table 1.** Dose/fractionation and BED of SRS and SBRT.

SRS/SBRT	Dose/fraction (fr)	Median BED_10_ (range)
1. SRS (*n* = 49)		
Brain oligometastases		
Lesion size		
<3 cm lesion	18-24 Gy/1 fr	70.4 Gy (60-81.6)
>3 cm lesion	24-36 Gy/5-6 frs	59.5 Gy (43.2-79.2)
2. SBRT (*n* = 39)		
Site of oligometastases		
Spine	24-35 Gy/3-5 frs	46.5 Gy (43.2-59.5)
Non-spine bone	30-35 Gy/3-5 frs	59.5 Gy (45-60)
Liver	35-55 Gy/3-5 frs	107.7 Gy (59.5-124.8)
Lung	50 Gy/5 frs	100 Gy (nil)

Abbreviations: BED = biologically equivalent dose; SRS = stereotactic radiosurgery; SBRT = stereotactic body radiotherapy.

Patients were followed up at 2-3 months interval with clinical examination, Contrast-enhanced computed tomography (CECT)/contrast MRI, and or PET-CT scan as required. Response assessment for SRS was done with Response Assessment in Neuro-Oncology Brain Metastases (RANO-BM) criteria and for SBRT combined radiological features with functional MRI and CT-PET.

### Statistical analysis

The statistical analysis was performed using IBM SPSS Statistics 2022. For patient outcomes, conventional descriptive statistical methods were used. Overall survival (OS) and progression-free survival (PFS) curves were estimated by the Kaplan-Meier method. Subgroup analysis for the impact of variables was done by log-rank test and chi-square test.

## Results

A total of 43 patients received SBRT/SRS to 88 metastatic lesions in addition to systemic therapy. Site of primary tumour and demographic data is shown in Table 2. The entire cohort of patients was evaluated together and then separately for patients with C and EC oligometastases. The median time to develop oligo-progression was 19.5 months (7-288 months).

**Table 2.** Demographic data.

Demographic data (*n* = 43)		
Age	Median 62 years (range 29-82 years)	
Gender	Male = 22 (51.2%)	Female = 21 (48.8%)
Primary site	NSCLC = 15 (34.9%)	
	Carcinoma breast = 14 (32.6%)	
	Carcinoma prostate = 5 (11.6%)	
	CRC = 5 (11.6%)	
	RCC = 4 (9.3%)	
Site of metastases	Cranial = 18 (41.8%)	
	Extra-cranial = 23 (53.4%)	
	Cranial and extra-cranial = 2 (4.6%)	
Number of patients with oligometastases	13/43 (30.3%)	
Number of patients with oligo-recurrence	30/43 (69.7%)	
Number of metastases treated	Cranial = 49 (SRS)	
	Extra-cranial = 39 (SBRT)	
	Bone = 28	
	Liver = 9	
	Lung = 2	
Number of patients received systemic treatment	40/43 (93%)	

Abbreviations: NSCLC = non-small cell carcinoma; CRC = colorectal carcinoma; RCC = renal cell carcinoma; SRS = stereotactic radiosurgery; SBRT = stereotactic body radiotherapy.

Overall, 23 patients received SBRT, 18 patients received SRS, and 2 patients received both (Table 3). 40/43 patients [93%] had received systemic therapy which included chemotherapy, hormonal therapy, IT (pembroluzumab, nivolumab), targeted therapy (cetuximab, tyrosine kinase inhibitors, herceptin, sunitinib, bevacizumab) or a combination as indicated.

**Table 3.** Outcomes of patients with cranial and extra-cranial oligometastases.

	Cranial (C)	Extra-cranial (EC)	C + EC
Number of patients	18	23	2
Age (years)	64 (38-82)	58 (29-73)	61 (58-64)
Primary tumour	NSCLC = 9 (50%)	NSCLC = 6 (26%)	Breast = 2
	Breast = 6 (33.3%)	Breast = 6 (26%)	
	RCC = 3 (16.6%)	Prostate = 5 (21.7%)	
		CRC = 5 (21.7%)	
		RCC = 1 (4.3%)	
Median follow-up	11 (0-48 months)	18 (0-60 months)	
Local control of SRS/SBRT	13/14 (92.8%)	19/22 (86.3%)	2/2 (100%) (*P* = 0.61)
Not known	4	1	
Local failure	1/14 (7.14%)	3/22 (13.6%)	*P* = 0.61
Distant failure	12/14 (85.7%)	7/20 (35%)	*P* = 0.03
Cranial	3	1	
Extra-cranial	4	5	
C + EC	5	1	
Not known	4	3	
Number of deaths	5 (27.7%)	5 (21.7%)	1 (50%)
PFS	Median 6 months	Median 30 months	*P* = 0.003
	SE: 1.25 (95% CI, 3.55-8.44)	SE: 8.45 (95% CI, 15.9-44.09)	
	1 year = 21.4% (SE: 0.110)	1 year = 73.7% (SE: 0.102)	
	2 years = 14.3% (SE: 0.094)	2 years = 53% (SE: 0.150)	
OS	Median 24 months	Median not reached	*P* = 0.211
	1 year = 77.6% (SE: 0.116)	1 year = 89.6% (SE: 0.070)	
	2 years = 48.5% (SE: 0.181)	2 years = 82.7% (SE: 0.091)	

Abbreviations: NSCLC = non-small cell carcinoma; RCC = renal cell carcinoma; CRC = colorectal carcinoma; SRS/SBRT = stereotactic radiosurgery/stereotactic body radiotherapy; PFS = progression free survival; OS = overall survival.

**Figure 1.**
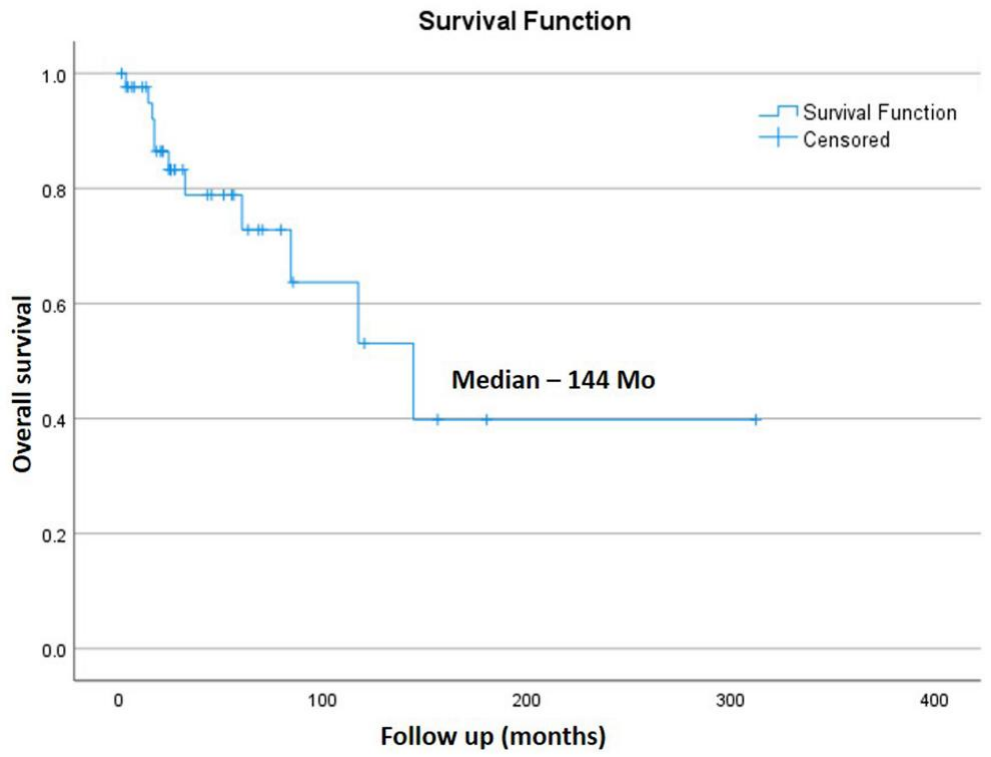
Overall survival of patients with oligometastases since diagnosis. Abbreviation: Mo = months.

**Figure 2.**
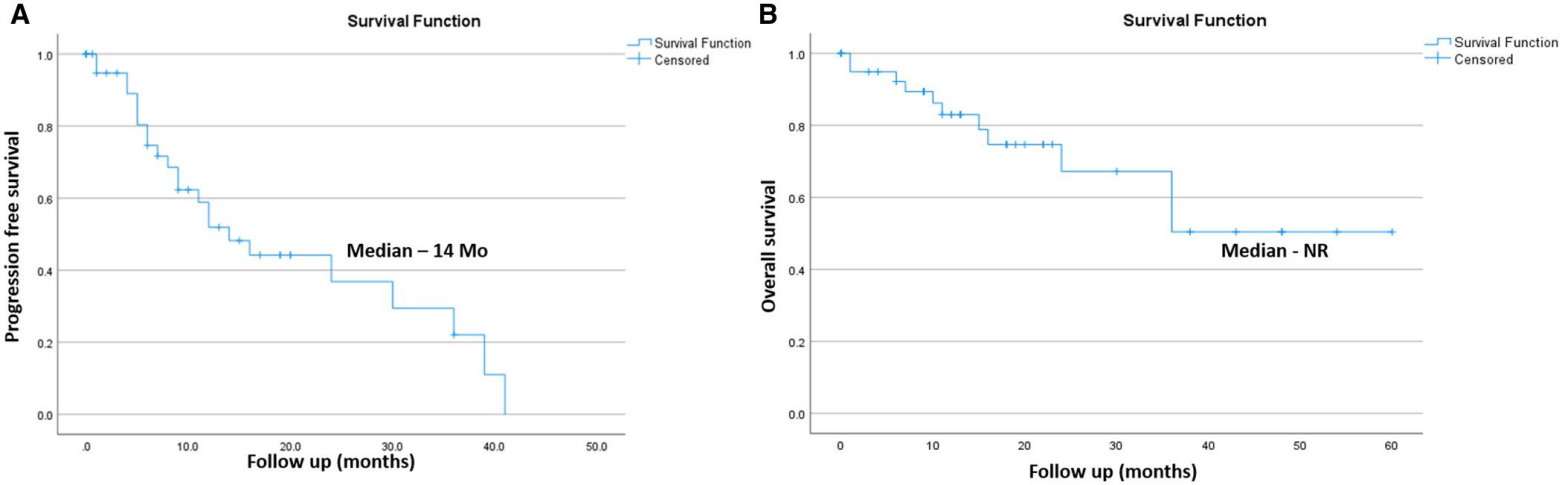
Survival of patients with oligometastatic disease—post-SRS/SBRT. (A) Progression-free survival; (B) overall survival. Abbreviations: SRS/SBRT = stereotactic radiosurgery/stereotactic body radiotherapy; NR = not reached; Mo = months.

Median follow-up of the entire cohort was 27 months (11-312 months) from diagnosis of the primary tumour. Eleven patients had died and 32 were alive till the time of assessment. The median OS of the entire cohort was 144 months and 1 and 2 years OS was 97.6% and 83.3% (Figure 1). Median follow-up after the detection of OMD was 13 months (0-60 months). 1 and 2 years PFS was 58.8% and 36.7% and OS was 83.2% and 67.4% (Figure 2). Overall local control with SRS/SBRT was 88.8% (Table 3).

Median PFS according to primary site was 41 months, 14 months, 12 months, 8 months, and 6 months for prostate, breast, non-small cell lung cancer (NSCLC), colorectal carcinoma (CRC), and renal cell carcinoma (RCC; *P* = 0.07). One and 2 years OS for breast cancer was 85.1% and 68.1%, for NSCLC 91.7% and 61.4%, for prostate cancer 100%, for RCC 66.7%, and for CRC 60% (*P* = 0.568).

### C vs EC metastasis

**Figure 3.**
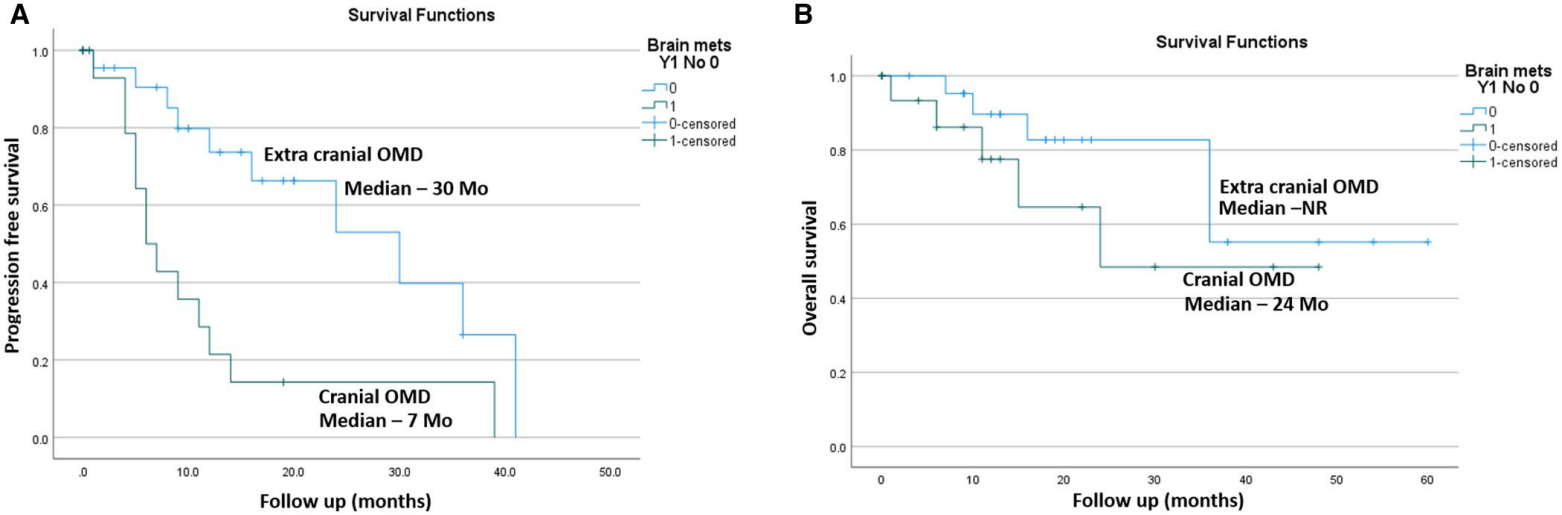
Survival of patients with cranial vs extra-cranial OMD. (A) Progression-free survival; (B) overall survival. Abbreviations: OMD = oligometastatic disease; NR = not reached; Mo = months.

Demographic details and outcomes of patients of C and EC oligometastases is shown in Table 3. Median PFS was 6 months for C metastases and 30 months for EC metastases (*P* = 0.003). 1 and 2 years PFS and OS for the 2 groups are shown in Table 3 and Figure 3.

### Bone vs visceral metastasis

Median PFS was 30 months for bone metastases and 7 months for visceral metastases (*P* = 0.001). 1 and 3 years OS was 93.8% and 56.8% for bone metastases and 74.6% and 49.8% for visceral metastases (*P* = 0.211).

### Local and distant relapse

For the entire cohort, response rate with SRS/SBRT was complete response in 25 patients, partial response in 6, stable disease in 3, progressive disease in 4, and unknown in 5. Local failures were higher for EC-OMD than C-OMD, but it was not statistically significant (*P* = 0.61). However, distant failures were significantly higher in C-OMD as compared to EC-OMD (85.7% vs 35%, *P* = 0.03; Table 3).

Seven patients (16.3%) eventually developed poly-metastases with estimated 1 and 3 years poly-metastases free survival of 88.2% and 72.8%.

### Toxicity

SBRT was well tolerated by majority of patients. One patient suffered grade 3 myelopathy (CTCAE v5.0) after re-irradiation with SBRT to cervical spine. Grade 1 toxicity was seen in 3/43 (6.97%) patients in the form of pain flare (1) and fatigue in 2 patients with SBRT to liver.

## Discussion

There is considerable divide among treating oncologists regarding management of OMD. A study reported notable divergence among oncologists regarding variability in treatment recommendations.^14^ A significant proportion of patients have OMD when the primary tumour metastasizes. Also, patients are increasingly being diagnosed with OMD due to advances in imaging technologies like CT-PET and systemic therapies which allow patients to live longer.^15^ Nearly 50% of patients with NSCLC, 43%-47% with breast cancer, and 41% with prostate cancer had 3 or less sites of metastases detected during their course of illness.^16–18^ However, the prognosis of patients with metastatic breast cancer treated with systemic therapy is dismal with median OS of 22.2 months and time to treatment failure of 6 and 10.5 months.^19^^,^^20^ For patients with metastatic NSCLC, median OS of 7.9 months with 4 different chemotherapy doublets was reported.^21^

Benefit of adding RT to oligo metastatic sites has been reported in many studies.^4^^,^^5^^,^^6^^,^^9^^,^^16^ A recent trial (Stereotactic Ablative Radiotherapy for the Comprehensive Treatment of Oligometastatic Tumor (SABR-COMET)) had reported median PFS of 12 vs 6 months (*P* = 0.001) and OS 41 vs 28 months (*P* = 0.09) with the addition of SBRT to OMD over standard of care alone.^4^ Milano et al reported outcome in 48 patients with OMD treated with SBRT and systemic therapy. It reported 5 years OS of 83% for bone-only OMD vs 31% for non-bone OMD and local control of 100% and 73%, respectively.^6^ Another retrospective study of EC-OMD treated with SBRT and systemic therapy reported 2 years PFS of 32%, OS 70%, and local control of 86.6%.^22^ Few other studies have reported local control rates of 70%-95% with SRS/SBRT, low toxicity, feasibility in majority of sites of metastases, and had unique ability to induce an immune response.^7–9^ Response rates of SBRT/SRS, PFS, and OS in our study are in conformity with these results.

As new data emerge, IT is finding a significant role in the treatment of many solid tumours. Its efficacy is variable due to variable immunogenicity and mutational burden. Some tumours are less sensitive to IT (cold tumour) due to dearth of T-cell infiltration compared to “hot” tumours.^23^ RT especially hypofractionated RT like SBRT can trigger the secretion of cytokines and chemokines that recruit immune cells into the tumour, potentially increasing anti-tumour immune surveillance.^23^^,^^24^ A phase II study on stage IV NSCLC with or without SBRT prior to Pembrolizumab showed higher median OS with combined therapy.^25^

A review of safety of combination of radiation therapy and various systemic agents has been reported by Guinod et al.^26^ Variable period of gap is suggested between RT and systemic therapy—3 days for BRAF and MEK inhibitors (like vemurafenib and dermafinib), 7 days for cetuximab, 1-2 days with tyrosine kinase inhibitors like geftinib/osimertinib, 4 weeks for VEGF (vascular endothelial growth factor) inhibitors like bevacizumab, 5-10 days for sorafinib and sunitinib and 2 days for IT with ipilimumab. For Her-2neu target therapy, no gap is required while for Lepatinib and TDM1, there is insufficient data.^26^ A potential risk of radiation necrosis with the combination of SRS and IT needs to be considered in patients with brain metastases.^27^

Furthermore, upfront systemic treatment with drugs crossing BBB such as crizotinib, alectinib, ceritinib, and osimertinib has been reported for patients with asymptomatic brain metastases and RT was offered in case of tumour progression.^28^^,^^29^

In the present study, median OS after the detection of OMD was not reached while median PFS was 14 months (Figure 3) as compared to SABR COMET trail which reported median OS and median PFS of 41 months and 12 months, respectively.^4^ However, this study has few differences compared to our study. Systemic therapy was given in 55% of patients vs 93% in the present study, brain metastasis was present in 1/66 patients vs 20/43 (46.5%) in our study, and patients with carcinoma prostate were 21% vs 11% in our study. Local control with SBRT/SRS was 88.8% in the present study as compared to 75% reported by Palma et al. However, the present study is retrospective, and study by Palma et al was prospective, phase 2 trial. The present study observed grade 3 toxicity of 2.32% and grade 1 of 6.97% as compared to grade ≥2 toxicity of 29% and grade 3 in 4.2% patients in the study by Palma et al.^4^ In another phase 2 trial—“SABR-5”, local control with SABR was 87% at 3 years and PFS rates at 1 year and 2 years were 56% and 31%.^30^ These results are comparable to the results obtained in the present study.

Patient with C-OMD are likely to have different clinical course as compared to patients with EC metastases since most of the chemotherapy drugs including many newer targeted drugs do not cross BBB or cross in insufficient concentration.^12^ Also there is no clarity on differences in the outcome in the 2 groups in terms of new sites of failure, PFS, and OS. In our study patients with C-OMD faired worse than EC-OMD (Table 3). Few other factors which could have contributed to poorer outcome of C-OMD included site of primary tumour. Fifty percent patients of C-OMD had primary NSCLC compared to 26% with EC-OMD, which has poorer prognosis compared to other primary sites (Table 3). Another factor can be biologically equivalent dose (BED_10_) delivered with SRS and SBRT. A co-relation of higher BED on LC and survival is reported for EC-OMD. A minimum BED > 75 Gy was found to have a favourable impact on OS, PFS, and local control as compared to BED < 75 Gy.^5^ However, BED is limited by the maximum achievable dose due to radiation tolerance of metastatic sites, eg, brain and spinal cord. For SRS, LC of >70% is achievable with BED_10_ of 40-50 Gy and LC of 80% with 50-60 Gy with risk of radiation necrosis below 2%-14%.^31^

In the present study, local control with SRS/SBRT was 88.8% which is comparable to the current data.^7–9^ Also systemic therapy was given to 93% of patients which would have made greater impact on outcomes of EC-OMD as compared to C-OMD. However, few studies have shown improved outcome if brain metastasis is treated with surgery or SRS in addition to the treatment of primary tumour.^32^

While there is considerable data showing benefit of adding SBRT to metastatic sites in EC-OMD, there is paucity of data on patients with C-OMD treated with SRS.^4–6^ Also many patients had received whole brain RT instead of SRS which yielded inferior OS and intra-C PFS as compared to SRS or surgery.^33^ The impact of co-existing EC disease in patients of C-OMD is largely unreported. One study had reported improved survival in brain-only OMD compared to patients with EC involvement (11 months vs 6 months).^34^ Another study had included patients with either no EC metastases or limited to 1 site.^35^

Similarly, most of the studies on EC-OMD have either excluded or have low proportion of patients with brain metastases.^4^^,^^9^^,^^30^ In the present study, patients with C oligometastases faired worse than the patients with EC metastases: (i) Median PFS was 6 months for C vs 30 months for patients with EC-OMD (*P* = 0.003); (ii) Median OS was 24 months for C-OMD and not reached for EC-OMD (*P* = 0.21); (iii) More patients with C-OMD suffered distant failures compared to EC-OMD (85.7% vs 35%, *P* = 0.03) including higher relapses in the brain (Table 3).

However, one of the limitations of our study is smaller number of patients and future studies with more number of patients would further help in understanding the differences between the 2 groups. Other limitation was the timing of SRS/SBRT in relation to systemic therapy. This aspect needs further validation according to the present and some of the ongoing studies.^26^^,^^27^

Patient selection is crucial and some of the factors reported to affect the outcome are disease-free interval (DFI) of >2 years from diagnosis,^3^ patients with primary in the breast, kidney, or prostate, DFI >75 months, ≤2 metastases, absence of liver, adrenal or mediastinal/hilar lymph nodal disease,^5^ bone only metastasis vs visceral metastasis.^6^ In agreement with above study,^6^ our study showed better mPFS for patients with bone metastasis compared to visceral oligometastases (30 months vs 7 months; *P* = 0.001). Median OS was not reached for bone metastases vs 24 months for visceral metastases (*P* = 0.211).

Effective ablation of OMD is reported to delay progression to poly-metastatic state with possible impact on OS. In patients of prostate cancer on hormone therapy, 5 years poly-metastasis free survival was 56% with SBRT vs 20% without SBRT.^36^ In the present study overall poly-metastases free survival at 1 and 3 years was 93.4% and 88.7% indicating significant delay in progression to widespread metastases.

Higher single-dose radiation therapy (SDRT) 24 Gy/1 fr to oligometastatic lesions has reported superior LC and improved distant metastatic progression.^36^ It has been postulated to work by dual mechanism involving microvascular vasoactive dysfunction and repression of homology-directed repair of DNA damage.^37^ Single fraction SRS for C metastases is well established and 13 patients in the present study received single fraction SRS. However, adoption of single fraction SBRT for EC-OMD has been limited among many institutions and studies.^3–6^ It may be due to lack of familiarity with the dose regimen and expected toxicity, especially in organs with significant motion like lung and liver. In the above-mentioned study, single fraction was delivered to bones, lymph nodes, and soft tissue deposits only which have least motion management issues.^36^ Furthermore, single-fraction SBRT in patients with prior thoracic radiation and/or chemotherapy has been reported to have higher toxicity.^38^ In the present study, SBRT to EC-OMD was delivered in 3-5 fractions with acceptable local control and toxicity.

Higher dose of SBRT and its integration with newer targeted drugs and IT have the potential to further improve the survival of these patients.

## Conclusions

SBRT and SRS are effective modalities for treatment of oligometastatic lesions with low toxicity. As compared to EC oligometastases, patients with C oligometastases had poorer outcome and frequent failures at new metastatic sites, including brain. Among EC oligometastases, patients with bony metastases faired better than visceral metastases.

This study suggests that patients with C and EC-OMD should be separately enrolled for future studies to further understand their long-term outcomes and rationalize their management.
